# Internet Addiction among Undergraduate Medical Students of a Medical College: A Descriptive Cross-sectional Study

**DOI:** 10.31729/jnma.7548

**Published:** 2022-06-30

**Authors:** Pravakar Dawadi, Sabina Khadka, Swojay Maharjan, Aashish Baniya, Sulochana Khadka, Sajina Thapa, Rajeeb Deo

**Affiliations:** 1 Nepalese Army Institute of Health Sciences, Sanobharyang, Kathmandu, Nepal; 2 Department of Medicine, Nepalese Army Institute of Health Sciences, Sanobharyang, Kathmandu, Nepal

**Keywords:** *internet addiction*, *medical students*, *Nepal*, *prevalence*

## Abstract

**Introduction:**

Internet addiction denotes the compulsive use of the internet which affects physical, mental, social, psychological and academic aspects of life of an individual. Very few studies among medical students in regard to internet addiction have been conducted. Our study aimed to determine the prevalence of internet addiction among undergraduate medical students of a medical college.

**Methods:**

This is a descriptive cross-sectional study conducted in a medical college from September to December, 2021 after obtaining ethical clearance from the Institutional Review Committee (Registration number: 442). The study was conducted among 229 medical students using convenience sampling technique. Internet addiction test questionnaire was used for collecting data. Data were entered in Google Spreadsheet and analysed with Microsoft Excel 2016. Point estimate at 95% Confidence Interval was calculated along with frequency and proportion for binary data.

**Results:**

The prevalence of internet addiction among 229 undergraduate students was found to be 121 (52.84%) (43.95-61.73 at 95% Confidence Interval). Out of them, mild and moderate internet addiction accounted for 90 (74.38%) and 31 (25.62%) respectively.

**Conclusions:**

The prevalence of internet addiction in this study was higher in comparison to the other studies conducted in similar settings.

## INTRODUCTION

Internet addiction simply refers to an individual's inability to control his or her use of the internet (including any online-related, compulsive behaviour) which can adversely affect their cognitive functioning, can lead to poor academic performance and engagement in risky activities.^1^

The risk of college students being addicted to the internet is rising particularly because of their psychosocial and environmental characteristics, unsupervised access to the internet and independent control of their time.^2,3^ A study conducted 5 years back among undergraduate students in Nepal showed the prevalence of internet addiction to be 35.4%.^4^ The burden of internet addiction is high among students; hence it is essential to quantify the burden and to adopt the necessary precautions on time to prevent the harmful consequences of internet addiction.

This study aimed to find out the prevalence of internet addiction among undergraduate medical students of a medical college.

## METHODS

A descriptive cross-sectional study was conducted among the first to fourth-year undergraduate medical students of the Nepalese Army Institute of Health Sciences-College of Medicine (NAIHS-COM) from September to December 2021. The study was conducted after receiving ethical approval from the Institutional Review Committee, NAIHS, in August 2021 (Registration number: 442). The students present in the active academic record were included in the study. Whereas, the students removed from the active database of academic records of the college who left the academic activity in between and those not giving the complete data were excluded from the study. Convenience sampling method was used. The sample size was calculated using the following formula:


$$\begin{array}{ll} \text{n} & ={\text{Z}}^{2}\times \frac{\text{p}\times \text{q}}{{\text{e}}^{\text{2}}}\\ & =1.96^{2}\times \frac{0.369\times 0.631}{0.05^{2}}\\ & =358 \end{array}$$

Where,

n = minimum required sample size
Z = 1.96 at 95% Confidence Interval (CI)
p = prevalence of internet addiction among medical students, 36.9%^5^
q = 1-p
e = margin of error, 5%

The above same size was calculated adjusted for finite population as:


n'=n1+n−1N=3581+358−1416=193

Where,

n'= adjusted sample size for finite population
n = total number of students from first to fourth year, 416

Taking 10% as a non-response rate, the minimum sample size required becomes 213. However, a total of 229 students took part in this study.

A standard and validated questionnaire developed by Dr Kimberly S. Young in 1998, the Internet Addiction Test was used for data collection.^7^ The questions present in the internet addiction test were re-typed in the Google form and the form was distributed to the selected study participants online. There were 20 questions for which the responses were recorded on the 5-point Likert scale (as 0, 1, 2, 3, 4 and 5).

The total score for a particular study participant was calculated as the sum of the responses provided on each question. The maximum score is 100 points.

To determine the internet addiction and its degree among the study participants, scoring system was used to classify the participants between normal, mild, moderate and severe internet addiction.^7^

Participants having scores either in mild, moderate and severe categories were considered to have some form of internet addiction. All the participants responded to the questionnaire.

Data were entered in the Google spreadsheets and later analysed in Microsoft Excel 2016. Point estimate at 95% Confidence Interval was calculated along with frequency and proportion for binary data.

## RESULTS

**Figure 1:**
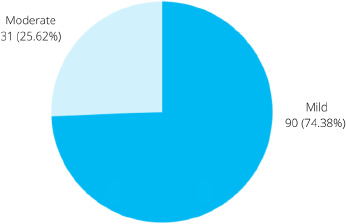
Degree of mild and moderate internet addiction (n= 121).

Among 229 study participants, the prevalence of internet addiction among the first to fourth-year undergraduate students of NAIHS-COM is found to be 121 (52.84%) (43.95-61.73 at 95% Confidence Interval). No student was found to have a severe level of internet addiction. Out of 121 participants having internet addiction, 90 (74.38%) and 31 (25.62%) were found to have mild and moderate internet addiction respectively (Figure 1).

Out of total 121 internet addicted students, 18 (14.88%), 30 (24.79%), 17 (14.05%) and 25 (20.66%) were having mild internet addiction among first, second, third- and fourth-year medical students respectively. Similarly, 7 (5.79%), 7 (5.79%), 10 (8.26%) and 7 (5.79%) students were having moderate internet addiction from first, second, third- and fourth-year medical students respectively (Table 1).

**Table 1:** Distribution of internet addiction among first to fourth-year medical students (n = 121).

Year in medical school	First year n (%)	Second year n (%)	Third year n (%)	Fourth year n (%)
Mild internet addiction	18 (14.88)	30 (24.79)	17 (14.05)	25 (20.66)
Moderate internet addiction	7 (5.79)	7 (5.79)	10 (8.26)	7 (5.79)

Among medical students having internet addiction, 55 (45.45%) male and 34 (28.10%) female medical students were found to have mild internet addiction. One (0.83%) student did not prefer to disclose their gender among student having mild internet addiction. Similarly, 22 (18.18%) male and 9 (7.44%) female medical students were found to have moderate internet addiction (Table 2). Out of 121 internet addicted participants, 77 (63.64%) were males and 43 (35.54%) were females.

**Table 2:** Gender distribution among participants having internet addiction (n = 121).

Gender	Male n (%)	Female n (%)	Prefer not say n (%)
Mild internet addiction	55 (45.45)	34 (28.10)	1 (0.83)
Moderate internet addiction	22 (18.18)	9 (7.44)	-

## DISCUSSION

The present study investigated the prevalence of internet addiction among medical students of a medical college. We found that 52.84% of students have some form of internet addiction. Among them, 74.38% had mild addiction, whereas 25.62% had moderate addiction. Comparable findings can be observed from a study in Western Maharashtra, India, with a prevalence of 58.87% internet addiction among undergraduate medical students where 87.35% were having mild and 12.65% had moderate internet addiction out of the overall internet addicted participants.^2^ The study reported no students having severe internet addiction, similar to our study. Another study also observed similar kind of pattern where 42.7% had internet addiction; out of whom 82.04% had mild, 17.37% had moderate and 0.59% had severe addiction among the college students attending professional courses.^8^

According to a study done among medical students in Iran, only 8% had moderate internet addiction among medical students of a university.^9^ Similarly, 18.01% of participants were experiencing moderate internet addiction among the ones who experienced internet addiction in another study among Greek medical students.^10^ Whereas, 11 .5% were problematic users having IAT score more or equal to 50 in a study conducted among Chilean medical students.^10^ The findings are comparable to another study among medical students in Delhi where 18% of medical students were observed to have problematic internet use behaviour, having an IAT score of 50 or more.^12^ These studies resemble the finding of our study, where 25.62% of the students experiencing internet addiction were found to be moderately addicted. Whereas a study reports 47.4% mild and 38.1% moderate internet addiction among Iranian medical students, which is similar to the finding in another study among students of three medical faculties in a University of Egypt where 47.7% of participants experienced moderate internet addiction.^12,14^ The findings of mild internet addiction in these studies are low compared to our study and the findings of moderate internet addiction is high compared to our study with the reference of percentage prevalence of mild and moderate internet addiction among overall internet addicted participants. Although our study found no participants experiencing severe internet addiction, several studies reported that 0.2%, 2.8%, 6%, and 12.9% of medical students had severe internet addiction with an IAT score of more than or equal to 80 respectively.^6,9,10,13^ This variable prevalence of internet addiction among medical students could be attributed to a diverse sample of participants as well as variable internet facilities among different geographical regions.

According to the study conducted in southwestern Iran among medical students, female medical students were observed to have more mild and moderate internet addiction among total research participants.^13^ The study among medical students of a medical college in Nepal also presented a similar pattern where 41.53%, 41.53% and 4.61% of females had mild, moderate and severe internet addiction, whereas 38.46%, 41.53% and 1.53% of males were having mild, moderate and severe internet addiction respectively.^15^ Another study among medical students of a university in Saudi Arabia demonstrates more female participants under severe internet addiction whereas more addiction among male participants under other types of internet addiction.^16^ Whereas in our study, 63.64% and 35.54% of participants addicted to internet were males and females respectively.

On the other hand, the study done in India shows more males were among internet addicted participants out of total participants.^2^ Similarly, 17.9% of males and 7% of females were addicted to the internet in another study done in Iran.^9^ Other studies report a similar pattern where a relatively high prevalence of internet addiction is recorded among male than female medical students with respect to the total number of participants enrolled in the study.^10,12,14^

The limitations of the study could be the response bias as well as recall bias from the participants. Since this study is a representative sample of medical students studying MBBS from only one medical college, the findings cannot be generalised to the other medical students.

## CONCLUSIONS

The prevalence of internet addiction in this study was higher in comparison to the other studies conducted in similar settings. Hence, interventions such as raising awareness regarding the detrimental effects of internet addiction among medical students should be done.
